# Reply to “FAIR chemical structure in the Journal of Cheminformatics”

**DOI:** 10.1186/s13321-021-00521-3

**Published:** 2021-07-07

**Authors:** Rajarshi Guha, Nina Jeliazkova, Egon Willighagen, Barbara Zdrazil

**Affiliations:** 1 Vertex Pharmaceuticals, Boston, MA 02129 USA; 2 Ideaconsult Ltd, Sofia, Bulgaria; 3 Department of Bioinformatics-BiGCaT, NUTRIM School of Nutrition and Translational Research in Metabolism, Maastricht University, Maastricht, The Netherlands; 4 Division of Pharmaceutical Chemistry, Department of Pharmaceutical Sciences, University of Vienna, Vienna, Austria

Schymanski and Bolton [1] requests the adoption of a FAIR approach to improve the FAIRness (findability, accessibility, interoperability, reusability) of chemical structures in the Journal of Cheminformatics. Currently, our journal encourages open science practices, but at the same time does not provide much guidance on how to implement these practices. Because FAIR chemical data is important [2], we welcome the open standard proposal and intend to adopt it as recommendation to authors.

Specifically, we will update the author guidelines that authors can improve the FAIRness by opting-in to provide chemical structures in open data associated with our journal articles in this format. For submissions where data is already provided in a FAIR format, an index file in this format can be provided. Second, we will make reviewers aware of this new, optional open standard.

While we agree with the importance of FAIR data, we also foresee practical complications when making this obligatory for all articles. Particularly, for the Database article type, the proposal may be both redundant to existing editorial standards as well as problematic for really large datasets as used in, for example, Probst and Reymond [3].

As a result, rather than require, we strongly recommend that authors include structure data in open, machine readable formats and identifiers, and point towards Schymanski and Bolton [1] as a means of doing so.
